# Supplementary material for: Analysis of quantile regression for race time in standard distance triathlons
Source: PLoS One. 2024 Nov 25;19(11):e0313496. doi: 10.1371/journal.pone.0313496 (PMC11588204; doi:10.1371/journal.pone.0313496)
Supplement: Attachment — Submitted filename: PLOS_REV.pdf [file pone.0313496.s003.pdf]

Thank you for providing an opportunity to review the manuscript. The general idea is interesting and the presented results seem worth publishing and discussing. To some extent, this is the complementary paper for “A study of triathletes’ race strategies in different competition environments” published in Heliyon 2024, by the same first author. However, revisions are required. Most importantly, I believe the necessary improvements are doable, therefore I encourage the authors to put the work into improving the paper. Please find my suggestions below:

1) You cannot phrase that favorable river current or altitude improves performance - the times might be faster, but it does not mean that the performance was better. Certain conditions might have resulted in faster times, and that is what you have analyzed in terms of the environment. Consequently, rephrase the goal of the study in lines 68-70 and all the relevant sections in the paper. It will also affect some parts reg interpretation. Faster time does not equal better performance.

2) As you only examined well-trained triathletes (Continental Cups, World Cups, domestic highest-level races) with professional backgrounds why do you differentiate amateurs and professionals? They are all pros, however on different levels. See McKay et al 2022, Participants Classification Framework (DOI: 10.1123/ijspp.2021-0451) and apply it correctly.

3) What about wetsuit influence? With olympic distance (now it is officially called standard distance so consider changing the phrase…) I believe above 20 Celsius wetsuits are forbidden, see World Triathlon Competition Rules for clarification. This is an important factor and should be mentioned. Hopefully, it could be taken into account in the analysis, as the water temp is provided on triathlon.org, at least in the limitations section.

4) Were these draft legal or non-drafting races? Were the competition rules the same for international and domestic races? If yes, mention that.

5) The paper structure is not appropriate, the headlines are not prioritized correctly, and some paragraphs belong to other sections. Ie lines 110-115 are redundant here, if you want to state the focus of the study then do it in the introduction. The methods section should state the design of the study first (retrospective analysis of public data) and then, dig into the details.

In multiple places, you do not provide references and you state conclusions (in the abstract all the sections should be changed) that do not originate from the study results or references work.

6) I cannot agree with excluding run split from the analysis due to so-called multicollinearity. A correlation of 0.545 is (conventional approach) considered moderate and without taking run into consideration the results are not really useful.

7) You repeat the results multiple times in the discussion, this is not a goal of the discussion section. Refer to relevant work and provide context for the results that you have already

provided in the Results section. If you feel your result presentation is not clear see APA or AMA guidelines, for example.

8) If you underline the role of specific training and testing to the race demands you might refer to the following papers, dois below:

10.23736/S0022-4707.24.15921-X

10.1111/j.1468-2419.2007.00286.x

doi.org/10.3390/sports7050101

10.1123/ijsnem.2018-0256

9) I recommend proofreading with a native speaker or editor. Language needs some improvement in flow and readability. Even the current title is not grammatically correct, with no clear reason why some words begin with capitals or small letters.

Additionally, some minor suggestions:

Lines 39-45 - remove ie from brackets, redundant.
48 - reference needed
Table 1 title - It is not a race schedule, these are events included in the study…
227-228 - speculative, provide reference or delete
270-275 - speculative, provide reference or delete
276-277 - reference needed
284-287 - out of scope, maybe you might address air density and drag as a contribution towards faster overall splits? or no clear explanation?
298 - underline it is a singular example
303 - provide reference
306 - provide reference
307-313 - the whole paragraph is out of the scope of the study
326 - LimitationS
347-351 - redundant
353-358 - speculative and not originating from the results, rephrase or delete

multiple double spacing and occasional capital letters in the middle of the sentence

TO SUM UP: The manuscript requires major revisions to be considered for publication. However, as the study design is acceptable, the results are interesting, the topic is important, all the necessary improvements are possible.
