# Supplementary material for: Analysis of quantile regression for race time in standard distance triathlons
Source: PLoS One. 2024 Nov 25;19(11):e0313496. doi: 10.1371/journal.pone.0313496 (PMC11588204; doi:10.1371/journal.pone.0313496)
Supplement: Attachment — Submitted filename: Response to Reviewers.docx [file pone.0313496.s001.docx]

**Response to reviewers**

Dear Chris Harnish,

We appreciate you and the reviewers for dedicating your valuable time to reviewing our paper and providing valuable comments. Your insightful comments have led to potential improvements in the current version. The authors have carefully considered the comments and have made every effort to address each one of them. We hope that the manuscript, after careful revisions, meets our high standards. The authors welcome additional constructive comments, if there are any. All revisions are highlighted in blue color in the manuscript.

First of all, thank you for sharing the reviewer's comments with us. We appreciate the insightful feedback and the opportunity to address the concerns raised. We have addressed the concerns raised as follows:

We have incorporated two new sections, "Methods Selection" and "Model Building," within the "Data" part of our manuscript to clearly articulate our rationale for specific modeling choices and interpretations.

Some of these conclusions may seem counterintuitive, such as the positive effects of altitude and temperature. So, we have added more supporting literature in the discussion section, for example on lines 282-284.

Pre-cooling with water is indeed an important aspect to be considered and we have reviewed the literature on this subject. However, the reason that water pre-cooling is not considered in the current study is that data on this aspect is not readily available. We have included this as a limiting factor on line 367 in the manuscript. Thank you for pointing this out. We will carefully consider this issue and investigate it in future studies.

We have carefully studied your recent paper co-authored with others (DOI: 10.1249/MSS.0000000000003328) to gain a deeper understanding of how to explain the outcomes suggested by our models. The paper titled "Making History in One Hour: How Gender, Age, Technique, and Altitude Affect Hour Record Cycling" delves into factors influencing the hour record cycling performance, and it deserves broader visibility and recognition for its valuable insights. We have cited this paper as it significantly contributes to explaining the models and results presented in our study.

Once again, we appreciate the editor's guidance and support.

When submitting revision, we have addressed these additional requirements to meet Journal Requirements.

1. we ensure that our manuscript meets PLOS ONE's style requirements, including those for file naming.

2.We have stated what role the funders took in the study: "The funders had no role in study design, data collection and analysis, decision to publish, or preparation of the manuscript."

3.We have removed any funding-related text from the manuscript. The updated statement reads as follows: “This research was supported by the Fundamental Research Funds for the Central Universities (approval number: 21lzujbkytd009) and the Research Project of Gansu Provincial Department of Transportation, China: Mathematical Model Study on the Impact of Traffic Volume and Some Environmental Factors on the Performance of Ordinary National Highways in Gansu Province (Grants No. 2023-03).”

4.We have declared that we have no competing interests.

5.We have removed the Supplementary Material [Figures S1-S6] and uploaded it separately as a “Supporting Information” file type, with a note following each Supporting Information file.

Please let us know if any further adjustments are required.

Below, the comments of the reviewers are addressed point by point, and the revisions are indicated.

**Response to Reviewer #1**

Thank you very much for reviewing our paper and your valuable suggestions. The issues you raised are very important to us and we will try our best to explain and improve our research. Regarding the serious and less serious flaws in the statistical methodology of the paper that you have mentioned, we are willing to elaborate and explain each of them:

1. Thank you for highlighting this issue. Your question is invaluable, and we have endeavored to follow your suggestion and make attempts to address the problem. Despite our efforts to locate additional variable data, we have not yet identified highly suitable instrumental variables.

Regarding your concern, after conducting quantile regression, we performed correlation tests between the residuals and three endogenous variables ((swim time, bike time, and run time). Based on the results from the regression at the 0.5 quantile for men, the correlations between the residuals and swim time, bike time, and run time were -0.017, -0.072, and 0.039, respectively, all below 0.1. This low correlation holds true across the 0.1, 0.5, and 0.9 quantiles. Therefore, despite the presence of endogeneity, its impact on the regression results is minimal, suggesting the current results are reasonably accurate.

Additionally, we have acknowledged this endogeneity issue as a limitation of our study in the discussion section. Moving forward, we remain committed to addressing this challenge by focusing on finding appropriate instrumental variables in our future research. Your input has been crucial in shaping our approach, and we welcome any further suggestions or considerations you may have.

2. The "Methods Selection" section provides a detailed explanation of why quantile regression was chosen as the analytical tool for exploring the performance of top-level triathletes.

The reason we use quantile regression is that we want to focus on the performance of athletes at different levels through quantile regression. For example, the effect of the three split times versus the overall time may be different depending on the level of the athlete. Quantile regression allows for a more comprehensive analysis of the conditional distribution of the dependent variable, which provides insight into how the independent variable affects different parts of the distribution and can capture potentially nonlinear relationships (three split times with overall time).

On the other hand, the effect of environmental factors on an athlete's performance can also be related to the level of the athlete, where high level athletes may be able to cope with the environment and maintain their performance, whereas lower-level triathletes may be subject to changes in their performance as a result of the environment. Athletes may have outliers in their performance due to factors such as the environment. Quantile regression handles these issues better than OLS.

In summary, the overall race time data exhibit right-skewness and non-normality, precluding the use of OLS regression. Moreover, our primary interest lies in the performance of athletes at the top 10th percentile level. Quantile regression addresses both these issues effectively, which is why we opted for this method.

3. The purpose of comparing the performance differences between male and female athletes was to determine if there is a significant difference. If there is a substantial difference, it may not be appropriate to treat them as a single population. Thus, subsequent experiments and discussions were conducted separately for males and females.

We did not assume or expect the variables to follow a normal distribution. The normality test was performed to determine the appropriate method for testing the differences. The results indicated that the data did not follow a normal distribution, leading us to choose non-parametric tests to examine the performance differences between male and female athletes. If the data had met the assumption of normality, we would have used parametric tests suitable for normally distributed data. Therefore, the choice of non-parametric tests was not dependent on the assumption of normality. We started with the simplest statistical approach, considering the data did not follow a normal distribution. Hence, we did not utilize parametric methods such as t-tests or analysis of variance (ANOVA). Instead, we chose the commonly used non-parametric Mann-Whitney test to examine the performance differences between male and female athletes. Importantly, this test does not rely on the assumption of normality.

4. For the positive effects of temperature, we have added more supporting literature in the discussion section, for example, on lines 272-296. The influence of altitude on the performance of top 10% male and female triathletes is not significant. However, for male and female triathletes at the 0.9 quantile, we have substantiated and explained the positive impact of altitude based on our dataset and relevant literature in the discussion section.

**Response to Reviewer 2**

**Comment 1:** The title of the manuscript is in a way as if this study is cause-and-effect study while there is no intervention and the authors just investigated possible relationship or contribution of some factors to performance in Olympic distance triathlons. It’s misleading and I recommend the authors to consider this point.

**Our response:** We gratefully appreciate for your valuable comment. We appreciate the reviewer's concern regarding the potentially misleading nature of the title. We agree that the title might suggest a causal relationship study, whereas our research primarily investigates factors that may influence performance in the Olympic distance triathlon. We have revised the title accordingly to accurately reflect the scope of our study.

**Comment 2:** Line 47, there is no logical connection between the sentence “This study also focuses…” and the previous parts of the introduction.

**Our response:** Thank you for pointing out the coherence issue in the introduction. We acknowledge the issue highlighted regarding the lack of logical connection in the sentence "This study also focuses..." on line 47. We have thoroughly revised the introduction, and the sentence in question has been removed entirely.

**Comment 3:** The introduction does not follow a logical and coherent approach to introduce the main issue, present the current knowledge, indicate the gaps and novel aspects of the study, and finally form a concrete hypotheses or questions. I highly recommend the authors to reconstruct the introduction.

**Our response:** Thank you for your feedback. We appreciate your thorough review of the introduction. We have taken your suggestions into consideration and work on reconstructing the introduction to ensure it follows a logical and coherent approach. We have understood the importance of properly introducing the main issue, presenting current knowledge, indicating gaps and novel aspects of the study.

**Comment 4:** Line 95, please include the name of the official website form where the environmental data were collected.

**Our response:** Thank you so much for your careful check. We have indicated the name of the official website where the environmental data was collected on line 95.

**Comment** **5:** Line 106, what the authors means by “inconsistent time record” which was set as an exclusion criterion?

**Our response:** We thank the reviewer for the very interesting comment. The term "inconsistent time record" refers to cases where there are discrepancies or anomalies in the recorded times of the athletes. This could occur due to various reasons, such as errors in recording the results, issues with data uploading on the website, or other factors that may lead to inconsistencies in the reported times. For example, if the sum of the split times for three segments (swim, bike, and run) and the two transition times (T1 and T2) does not match the total time, or if the individual segment times deviate significantly from expected norms, it suggests that there might be issues with the data for that particular athlete's performance. As a result, these inconsistent time records are excluded from the dataset to ensure the integrity and reliability of the analysis.

**Comment** **6:** In Table 2, there is an inconsistency between total time and the time of each segment (swim, bike, and run).

**Our response:** We feel sorry for the inconvenience brought to the reviewer. Table 2 provides a descriptive statistic of the data, showing the maximum and minimum values for overall time in seconds, as well as the time for each segment (swim, bike, and run). However, it is important to note that the shortest total time does not necessarily correspond to the lowest time for each individual segment. Similarly, when the swim time is at its minimum, it does not mean that the athlete's overall performance is the best. This is because each segment's time is calculated independently, and the shortest total time simply reflects the combination of those individual times. Therefore, the inconsistency between the sum of segment times and the total time is a normal phenomenon.

**Comment 7:** Line 108, the Statistical Analysis section must include all the details related to any statistical tests used in the study. There are some missing points that should be considered.

**Our response:** Thank you so much for your careful check. For the statistical analysis section, we have added all the relevant details related to the statistical tests used in the study, e.g., line 155 and 162. Please let us know if there are any omissions.

**Comment 8:** Is investigating the difference between two sexes one of the study goals? Nothing has been mentioned in this regard.

**Our response:** Thank you for your feedback. Investigating the difference between the two sexes is not one of the study goals. However, it's important to address gender differences as they can impact the model's outcomes. If there are substantial performance variations between male and female triathletes, treating them as a single group for quantile regression may not be suitable. Therefore, we investigated the difference between two sexes before proceeding.

**Comment 9:** While most of the quantile regression studies are interested in finding the median of Y, why the authors set the Ƭ of 0.1th and 0.9th in the present study?

**Our response:** Thank you so much for your careful check. The selection of 0.1 and 0.9 quantiles instead of focusing solely on the median is based on the intention to capture extreme performance outcomes and assess the impact of factors at the lower and upper ends of the performance spectrum. Typically, most quantile regression studies focus on identifying the median of the dependent variable Y, which corresponds to τ=0.5. However, in the present study, set τ at 0.1 and 0.9 because we were interested in performance between top performers (top 10%) and average performers (top 90%, representing the majority of normal athletes) concerning the three transition times and environmental factors such as temperature, altitude, and water conditions.

**Comment 10:** In the results section, more detailed information regarding the order of influence of each independent variables for each quintile and also for sexes must be provided. All these information is missing in the results section. In the meantime, the authors have used such information in the discussion section without presenting them in the result section.

**Our response:** Thank you so much for your careful check. We have provided a detailed presentation of quantile regression results in the Results section, exemplifying the interpretation of findings using one specific quantile. In the Discussion section, we have meticulously analyzed and discussed the results in alignment with our findings.

**Comment 11:** In the discussion section, there are no strong support for the findings of the study. For example, the authors arguing that by increasing the altitude the performance increases and attributed this finding to acclimatization of the athletes. However, the mean altitude of races was 186 m above the sea level which normally has no considerable effect of body. In another example, the authors stated that the difference in the importance of running and swing as the predictors of the overall time is attributed to the difference in physical activity of athletes at 0.1 and 0.9 percentile. Why is that? No further information has been provided.

**Our response:** Thank you so much for your careful check. We have included more analysis and relevant literature support in the discussion section. All the relevant contents you have pointed out have been modified, specifically on the lines 308-318.

**Comment 12:** The main text of the manuscript needs a very careful revision in terms of the grammar, writing style, and verbs’ tense.

**Our response:** Thank you so much for your careful check. Your attention to these details is greatly appreciated. We have reviewed to ensure clarity and coherence throughout the manuscript. Your input has been crucial in shaping our approach, and we welcome any further suggestions or considerations you may have.

Thank you once again for your guidance throughout the review process. We look forward to hearing from you regarding the next steps.

Sincerely,

The Authors
