# Supplementary material for: Analysis of quantile regression for race time in standard distance triathlons
Source: PLoS One. 2024 Nov 25;19(11):e0313496. doi: 10.1371/journal.pone.0313496 (PMC11588204; doi:10.1371/journal.pone.0313496)
Supplement: Attachment — Submitted filename: Response to Reviewers.docx [file pone.0313496.s002.docx]

**Response to Reviewers**

Dear Chris Harnish,

We appreciate you and the reviewers for dedicating your valuable time to reviewing our paper and providing valuable comments. Your insightful comments have led to potential improvements in the current version. The authors have carefully considered the comments and have made every effort to address them. All revisions are highlighted in blue color in the manuscript.

In response to Reviewer #1's comments and the modifications made, we provide the following clarification:

We have revised the model as detailed in lines 141-156 of the manuscript. The model results are on lines 181-189. Specifically:

*1、Overall time (seconds) = Swim time (seconds) + Bike time (seconds) + Run time (seconds) + Transition 1 + Transition 2. Since transition times are not the focus of this study, they are not analyzed in the manuscript.*

*2、Given the correlation coefficient of 0.55 between Run time and Bike time, we have excluded Run time from the quantile regression model.*

*3、We included all performance data and introduced a gender dummy variable to expand the sample size, enhance the robustness of our results, and examine the effect of gender on triathlon performance.*

These changes address the issue of regressing overall time on the sum of swim, bike, and run times, thus avoiding coefficients close to 1. The discussion and conclusions of the revised model have been updated accordingly, as noted in lines 230, 257, 313, and elsewhere.

Regarding the use of instrumental variables, we attempted to use the water environment as an instrument for swim time. The water environment theoretically meets the requirements for an instrumental variable for swim performance (since it influences swim performance but does not directly affect the overall time). However, using the water environment alone did not yield satisfactory results, and the model performance was poor. Therefore, this approach was not adopted.

Reviewer’s points are valuable. We acknowledge that more data on suitable instrumental variables (e.g., sub-event training performance, physical tests, training duration) would be beneficial. However, we cannot access this data in the short term. We have discussed this limitation in the Discussion section and will consider it seriously for future research.

We hope that the manuscript, after careful revisions, meets our high standards. The authors welcome additional constructive comments, if there are any. Thank you once again for your guidance throughout the review process. We look forward to hearing from you regarding the next steps.

Sincerely,

The Authors
