# Supplementary material for: Analysis of quantile regression for race time in standard distance triathlons
Source: PLoS One. 2024 Nov 25;19(11):e0313496. doi: 10.1371/journal.pone.0313496 (PMC11588204; doi:10.1371/journal.pone.0313496)
Supplement: Attachment — Submitted filename: Response to Reviewers (1).docx [file pone.0313496.s004.docx]

**Response to Reviewers**

Dear Chris Harnish,

We are very grateful to you and the reviewers for your positive comments on the study and for the constructive comments you provided. We have carefully considered the suggestions of Reviewer #3 and have made the following revisions. All revisions are highlighted in blue color in the manuscript.

In response to Reviewer #3's comments and the modifications made, we provide the following clarification:

1) You cannot phrase that favorable river current or altitude improves performance - the times might be faster, but it does not mean that the performance was better. Certain conditions might have resulted in faster times, and that is what you have analyzed in terms of the environment. Consequently, rephrase the goal of the study in lines 68-70 and all the relevant sections in the paper. It will also affect some parts reg interpretation. Faster time does not equal better performance.

**Our response:** Thank you for your insightful feedback regarding the interpretation of performance in relation to environmental factors. We appreciate your clarification that faster times do not necessarily equate to better performance, as they may be influenced by favorable conditions such as river currents or altitude.

In response to your comments, we will revise the goal of the study stated in lines 83-92 and adjust all relevant sections throughout the paper to reflect this important distinction. We will emphasize that our analysis focuses on how specific environmental conditions may impact the recorded times without making direct claims about improvements in athletic performance itself. Additionally, we recognize that this adjustment will affect various parts of the interpretation in the paper, and we will ensure that our conclusions are consistent with this updated perspective.

2) As you only examined well-trained triathletes (Continental Cups, World Cups, domestic highest-level races) with professional backgrounds why do you differentiate amateurs and professionals? They are all pros, however on different levels. See McKay et al 2022, Participants Classification Framework (DOI: 10.1123/ijspp.2021-0451) and apply it correctly.

**Our response:** Thank you for your insightful feedback regarding the differentiation between amateurs and professionals in our study. Indeed, we examined well-trained triathletes (Continental Cups, World Cups, and domestic highest-level races) with professional backgrounds.

In our study, we focused on the performance records of the "Elite Group," as classified by the China Triathlon Association, which includes categories such as Elite, Youth, and U19. The emphasis on the "Elite Group" is due to the highly competitive ability of the athletes within this category, which provides more representative data for our analysis. This group comprises both male and female elite athletes, corresponding to the Elite Men and Elite Women categories on the official World Triathlon website. We have accurately described this in the manuscript, specifically in line 101-104 for clarity.

Regarding your reference to the study by McKay et al. (2022), it is important to note that due to the challenges of quantifying performance in triathlon, the original paper states: "In sports where quantifying performance isn’t appropriate (e.g., rowing, where environmental conditions and wind speeds can affect performance; and BMX/Mountain biking where races are completed on different courses), athlete ranking and placings at major competitions should be the priority metric used to classify athletes." Therefore, we have aligned our classification with the World Triathlon, which define the Elite Group as a professional triathlete who competes at an international level.

3) What about wetsuit influence? With olympic distance (now it is officially called standard distance so consider changing the phrase…) I believe above 20 Celsius wetsuits are forbidden, see World Triathlon Competition Rules for clarification. This is an important factor and should be mentioned. Hopefully, it could be taken into account in the analysis, as the water temp is provided on triathlon.org, at least in the limitations section.

**Our response:** Thank you for your valuable feedback regarding the influence of water temperature and wetsuits on the analysis of the triathlon events. We appreciate your insights into this important aspect.

In our study, we analyzed triathlon competitions held in China from 2013 to 2023, including the National Triathlon Championship Cup series. However, we found that many events prior to 2020 did not have recorded water temperature data on the China Triathlon Association website (http://triathlon.sport.org.cn/china/), which restricts our ability to analyze the effects of water temperature and the use of wetsuits.

We have clearly pointed out this limitation in the limitations section of our paper, demonstrating our commitment to data integrity and the rigor of our analysis. Furthermore, we plan to include the water temperature variable in future studies and will seriously consider its impact on competition results.

4) Were these draft legal or non-drafting races? Were the competition rules the same for international and domestic races? If yes, mention that.

**Our response:** Thank you for your valuable feedback! In the standard distance triathlon "Elite Group" races, drafting is allowed. This rule is consistent with the World Triathlon regulations, as specified in our manuscript on line 109-112.

Additionally, the competition rules are the same for both international and domestic races, ensuring consistency in how events are conducted. For further details, you can refer to the World Triathlon Competition Rules at the following link: [World Triathlon Competition Rules] (https://www.triathlon.org/uploads/docs/World_Triathlon_Sport_Competition_Rules_2020_201811253.pdf#:~:text=The World Triathlon Competition Rules are intended to: (i).

5) The paper structure is not appropriate, the headlines are not prioritized correctly, and some paragraphs belong to other sections. Ie lines 110-115 are redundant here, if you want to state the focus of the study then do it in the introduction. The methods section should state the design of the study first (retrospective analysis of public data) and then, dig into the details.

**Our response:** Thank you for your detailed feedback regarding the structure of the paper. We have adjusted the prioritization of the headings. Additionally, we have removed the redundant content in lines 110-115 as you suggested.

Regarding the missing references, we have carefully reviewed our conclusions to ensure that all statements are adequately supported by relevant literature. This can be found in the revised manuscript on lines 254, 257, and 299.

6) I cannot agree with excluding run split from the analysis due to so-called multicollinearity. A correlation of 0.545 is (conventional approach) considered moderate and without taking run into consideration the results are not really useful.

**Our response:** We appreciate your comments on the exclusion of the run split analysis, particularly considering that a correlation of 0.545 is deemed moderate. We have re-evaluated this decision and have decided to retain the run split in the regression analysis to enhance the practical utility of our results. The updated results can be found on line 179-182.

7) You repeat the results multiple times in the discussion, this is not a goal of the discussion section. Refer to relevant work and provide context for the results that you have already provided in the Results section. If you feel your result presentation is not clear see APA or AMA guidelines, for example.

**Our response:** The discussion has been carefully reviewed to eliminate any repetitive descriptions of the results. Relevant literature has been cited to provide additional context for the findings.

8) If you underline the role of specific training and testing to the race demands you might refer to the following papers, dois below:

10.23736/S0022-4707.24.15921-X

10.1111/j.1468-2419.2007.00286.x

doi.org/10.3390/sports7050101

10.1123/ijsnem.2018-0256

**Our response:** Thank you for your valuable feedback regarding the references to specific training and testing in relation to race demands. We appreciate your suggestions and have incorporated them into the manuscript. In the Introduction, specifically lines 60-72, we have now included the references to underline the importance of tailored training and testing protocols in addressing the demands of racing.

9) I recommend proofreading with a native speaker or editor. Language needs some improvement in flow and readability. Even the current title is not grammatically correct, with no clear reason why some words begin with capitals or small letters.

**Our response:** The manuscript has been thoroughly proofread to enhance flow and readability. Additionally, the title has been revised to ensure it adheres to grammatical standards.

We would like to express our sincere gratitude for the reviewer's helpful suggestions. We have carefully reviewed and made the necessary revisions, which are detailed below:

1. Lines 39-45: The term "i.e." has been removed as it was deemed redundant.
2. Line 48: A relevant reference has been added to support the statement.
3. Table 1 Title: The title has been revised to clarify that it lists events included in the study rather than a race schedule.
4. Lines 227-228 and 270-275: The speculative content has been deleted.
5. Lines 276-277: The content requiring a reference has been deleted in the revision.
6. Lines 284-287: This section, deemed out of scope, has been removed.
7. Line 298: The content has been deleted.
8. Lines 303 and 306: Both sentences requiring references have been removed from the manuscript.
9. Lines 307-313: This paragraph has been removed as it was out of the scope of the study.
10. Line 326: The typo in "LimitationS" has been corrected to "Limitations."
11. Lines 347-351: Redundant content has been deleted for conciseness.
12. Lines 353-358: Speculative content that did not originate from the results has been deleted.

We believe these modifications enhance the clarity and rigor of the manuscript. Thank you once again for your valuable suggestions, which have greatly contributed to our work. If further adjustments or additions are needed, please let us know.

Lastly, we hope that the manuscript, after careful revisions, meets our high standards. The authors welcome additional constructive comments, if there are any. Thank you once again for your guidance throughout the review process. We look forward to hearing from you regarding the next steps.

Sincerely,

The Authors
